# *STAG1*: Bridging the Gap Between Cohesin Complex and Epigenetic Machinery

**DOI:** 10.3390/genes17040483

**Published:** 2026-04-18

**Authors:** Tiziano Palazzotti, Giulia Bruna Marchetti, Rosa Maria Alfano, Ilaria Bestetti, Palma Finelli, Donatella Milani

**Affiliations:** 1 Department of Health Sciences, University of Milan, 20122 Milan, Italy; tiziano.palazzotti@unimi.it; 2 Fondazione IRCCS Ca’ Granda Ospedale Maggiore Policlinico di Milano, 20122 Milan, Italy; giulia.marchetti@policlinico.mi.it (G.B.M.); rosamaria.alfano@policlinico.mi.it (R.M.A.); ilaria.bestetti@policlinico.mi.it (I.B.); palma.finelli@policlinico.mi.it (P.F.); 3 Department of Pathophysiology and Transplantation, University of Milan, 20122 Milan, Italy

**Keywords:** neurodevelopmental disorder, *STAG1*, chromatinopathy

## Abstract

**Background**: The *STAG1* gene has been related to a poorly known form of intellectual disability, known as Intellectual Developmental Disorder, Autosomal Dominant 47 (MRD47). Functionally, MRD47 is part of the Cohesinopathies, a small family of rare genetic disorders caused by defective cohesin complex, whose activity is essential for sister chromatid cohesion and therefore for chromatin organization. Chromatin state modulation is an entangled process finely modulated by a large number of actors that, if altered, give rise to the so-called Chromatinopathies. The clinical and biological overlap among these families of conditions on one hand poses significant challenges during diagnostic definition, and, on the other, may help delineate more accurate management guidelines. **Methods**: Starting from the report of a novel pathogenic variant in the *STAG1* gene, we performed a retrospective clinical and molecular review of all previously reported patients affected by this rare disorder. Once clinical and photographic data of all published patients were collected, we used Face2Gene deep learning technology to analyze *STAG1* facial phenotype, comparing it to both Chromatinopathy and Cohesinopathy profiles. **Results**: Our clinical and molecular re-evaluation of reported cases confirms MRD47 as a mainly neurodevelopmental disorder. Through artificial intelligence technology, we were able to first create the gestaltic profile of MRD47. Face2Gene analyses of this composite phenotype, although limited by the tool’s analysis modalities, demonstrates the strong overlap of *STAG1* disorder with Chromatinopathies. **Conclusions**: The present literature review, together with gestaltic analyses of the *STAG1*-related phenotype, underscores the strong resemblance of MRD47 to epigenetic machinery disorders. The present case brings to light once more the biological and phenotypical entanglement of Cohesinopathies and Chromatinopathies, hinting at *STAG1* as the joining chain.

## 1. Introduction

Chromatin is composed of nucleic acids and proteins, whose interaction generates two compaction degree statuses: the open one (euchromatin), available for transcription, and the closed one (heterochromatin), unavailable instead [1]. The dynamic switching between these states is regulated by complex and fine-tuned mechanisms regulated by the wheels of the Epigenetic Machinery. Each epigene encodes a protein with a variable role in these processes: writers, readers, erasers and remodelers [2]. Chromatinopathies (CPs) are a large group of rare genetic conditions caused by a germline mutation in epigenes, resulting in chromatin state imbalance. To date, about 179 epigenes and relative CPs have been described [3]. CPs share common phenotypic features, including neurocognitive impairment, growth abnormalities, gestaltic anomalies and malformations.

Cohesin complex forms a ring within which sister chromatids can be trapped, allowing their correct segregation during mitosis [4]. It also participates in the spatial organization of DNA in the nucleus and regulates gene expression, crucial for neuronal development [5]. This complex is made by various subunits interacting with each other and with DNA; in particular, *SMC1*, *SMC3* and *RAD21* assemble to form a ring-shaped structure, tied by a STAG protein alternatively incorporating either STAG1 and STAG2 for each complex [6]. Mutations in the genes coding for cohesin complex members or cofactors cause developmental disorders, known as “Cohesinopathies” (CoPs), whose archetype is Cornelia de Lange syndrome (CdLS, #MIM 122470) [7].

*STAG1* (#MIM 604358, Cohesin Subint SA-1) encodes for a 1258-aminoacid-long protein with three different domains: the STAG domain (161–268) that binds the cohesion complex; the stromalin conservative domain (SCD) (296–381) that gives structural stability to the protein; and the HEAT-repeats domain (483–747) that interacts with regulators [8]. Pathogenetic variants in the *STAG1* gene cause “Intellectual Developmental Disorder, Autosomal Dominant 47” (MRD47, #MIM 617635), characterized by mild to moderate intellectual disability, behavioral issues and autistic features. Other clinical manifestations include microcephaly, defective growth possibly related to feeding difficulties, epilepsy and typical facial features [9]. To date, only 30 affected patients harboring 27 different variants have been reported in the literature [9,10,11,12,13,14,15,16,17,18].

Here, we describe a young girl carrying a novel *STAG1* pathogenic variant. The aim of the present work is not only to expand the current knowledge of this poorly characterized CoP but more importantly to characterize its gestaltic profile. To our knowledge, this is the first work extensively investigating through AI technologies the phenotypical overlap among *STAG1* disorder and CPs.

## 2. Case Report

The patient is a young girl who was first referred to the Pediatric Ambulatory of Genetic Diseases at the age of 10 for intellectual disability (ID). Her family history was unremarkable. Pregnancy and perinatal history were uneventful, though she was described as a “sleepy baby” with a mild brachycephaly. Developmental milestones were delayed: she took her first steps at 17 months, and she also had impairment in speaking and learning abilities. At 7 years, her Intelligence Quotient (IQ) score was 45, consistent with a moderate ID. Behavioral disorders (mainly characterized by sudden outbursts of anger against objects) emerged in late childhood and required pharmacological treatment. An MRI ruled out brain malformations. She also had mild hyperopia. Audiological, cardiological and endocrinological evaluations were normal.

**Figure 1:**
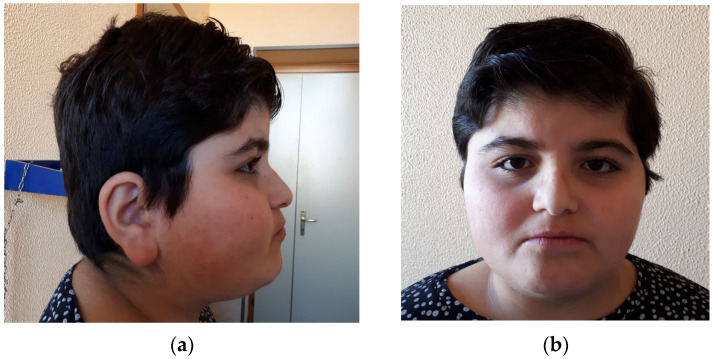
Gestaltic characteristics of our proband. (**a**) Left profile of the patient’s face; (**b**) frontal picture of the patient’s face.

According to her parents, her growth had always been regular until the age of 8, when she started to gain weight; she later developed hepatic steatosis. On our first evaluation her weight and head circumference (HC) exceeded the upper limits (+5 SD and +4 SD, respectively). We noticed some peculiar facial features: low anterior and posterior hairline; horizontal eyebrows; long eyelashes, downslanted, elongated and laterally everted palpebral fissures; convex nasal ridge with a low-hanging columella; thin lips; large pinnae; and fleshy lobes (see Figure 1a,b). We also noticed cervicodorsal hypertrichosis and carpal bone shortening.

Given the lack of specificity of her clinical picture, the first step into genetic testing was made through molecular karyotyping (array-CGH), which was normal. Driven by gestalt, the peculiar disposition of body hairs, the overgrowth and the ID, we required a trio clinical exome hypothesizing CPs. We identified a de novo heterozygous frameshift variant c.89_90delAG, p.(Glu30Glyfs*18) in the *STAG1* gene. The variant was newly described and classified as pathogenetic according to ACMG criteria (PVS1, PM2, PS2). Consequently, a diagnosis of MRD47 was established, a condition formally included among CoPs.

## 3. Materials and Methods

Once informed consent was obtained, DNA was extracted from leukocytes using the standard procedure, and trio clinical exome sequencing (CES) was performed. Briefly, the exonic regions and flanking splice junctions’ regions of the genome were captured using the Sure Select CD clinical Focused Exome (Agilent Technologies, Santa Clara, CA, USA), and sequencing was performed on a NextSeq2000 Illumina system (Illumina, San Diego, CA, USA) with 150 bp paired-end reads. Sequence reads were aligned to the reference human genome assembly (February 2009, GRCh37/hg19) and analyzed for variant calling and annotation using the Expert Variant Interpret—Evai V.3.6 (enGenome, Pavia, Italy) software. Rare single-nucleotide and indel variants were then filtered and prioritized based on population frequencies, pathogenic predicted effect, and inheritance. Selected variants were classified according to the ACMG guidelines [19]. The potential causative variant was subsequently confirmed by Sanger sequencing in the proband and parents using an independent DNA sample. A literature review was performed on Pubmed (accessed on 3 February 2026) looking for papers reporting clinical data of patients harboring pathogenic alterations of the *STAG1* gene (search terms: “STAG1 AND syndrome”). Out of 24 papers, we selected 7 studies that described clinical features associated with *STAG1* variants in humans [9,10,11,13,14,15,20]. Data collected from these 7 papers led to identification of 5 more articles detailing clinical data from affected individuals [12,16,17,18,21]. An in-depth review of the content led to the exclusion of papers or patients with dual diagnoses or harboring multi-gene deletion of locus 3q22 [9,14,21].

For the gestalt analysis we used the Face2Gene software (FDNA Inc., Boston, MA, USA; https://www.face2gene.com, version 26.1.0 accessed on 4 February 2026), freely available upon registration. In these deeper analyses we included not only our patient’s photos but also those of patients harboring a pathogenic *STAG1* point mutation or single gene deletion. Only frontal images were selected, and all pictures with any type of censorship were excluded. Thanks to an archive of photos available for research on patients treated at our Center, we ran a three-cohort research analysis to compare *STAG1* patients to both CoPs and CPs.

## 4. Results

### 4.1. Literature Data Review

**Figure 2:**
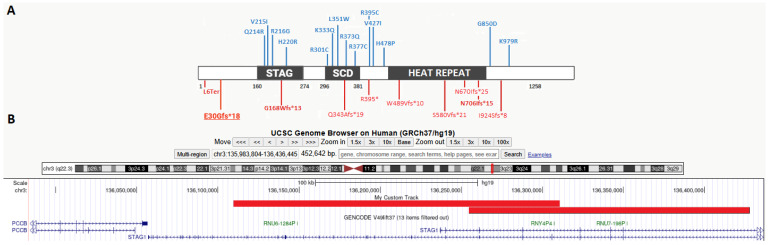
(**A**) Schematic representation of STAG1 protein and localization of causative variants reported up to date. Gray boxes represent functional domains of the protein (STAG domain and stromalin conservative domain, SCD, and HEAT repeat). Variants are reported with two different colors according to biological effect: red reports frameshift/truncating variants and blue reports missense variants. The variant described in the present patient is the one underscored. (**B**) Deletions reported by Lehalle et al., 2017 [9] involving exclusively the *STAG1* locus and considered for the present review.

A total of 12 English articles were retrieved, involving 30 individuals with pathogenic alterations of the *STAG1* gene. To accurately evaluate clinical features related to defective *STAG1* expression, we considered clinical characteristics of 27 patients harboring 23 different *STAG1* SNVs (detailed in Table 1) and two intragenic deletions of locus 3q22 (see Table 1). Figure 2 reports all 23 pathogenic point mutations reported in the affected cohort of patients. In detail, we collected 14 missense variants, mostly located inside functional domains (four within STAG and five within SCD respectively) and 10 frameshift/truncating alterations distributed throughout the gene.

Clinical features of the 27 patients considered in the present review are summarized in Table 2. Detailed description of each case can also be found in Supplementary Materials.

*STAG1*-related disorder globally emerges as a mainly neurodevelopmental condition with nearly all patients displaying some degree of cognitive impairment, often associated with behavioral difficulties and less frequently with autism spectrum disorder. Functional and morphological brain anomalies have been detected in almost one third of patients (36% and 28% respectively). Aside from facial traits that have been separately considered in this work, hand and foot anomalies appear to be quite common, including one major deformity (clubfoot) reported by Bregvadze and colleagues (2024). Congenital malformations represent occasional findings in *STAG1* patients but can virtually affect any apparatus (e.g., malformed ears and cleft lip and palate have been described). From an auxological perspective, both excessive and impaired growth have been reported across all the variables considered, including stature, weight, and head circumference (HC).

In addition to collecting photos of *STAG1* patients wherever available, we also recorded all reported facial features described for each patient (see Supplementary Materials). According to these data, most recurrent *STAG1* facial features include thin eyebrows (6 out of 27), deep-set eyes (16/18) and wide mouth (10/13).

### 4.2. Face2gene Gestaltic Analyses

Furthermore, we analyzed through Face2Gene single facial images of our case (N = 2) and of any MRD47 patients available from the literature (N = 8). Detailed results from these AI analyses can be found in the Supplementary Material. Using AI for gestalt evaluation, CdL emerges as the most recurrent one, being suggested among the top 3 in 4 photos out of 11 (10). Overall, CPs account for nearly one third of the top 10 suggestions (34%).

**Figure 3:**
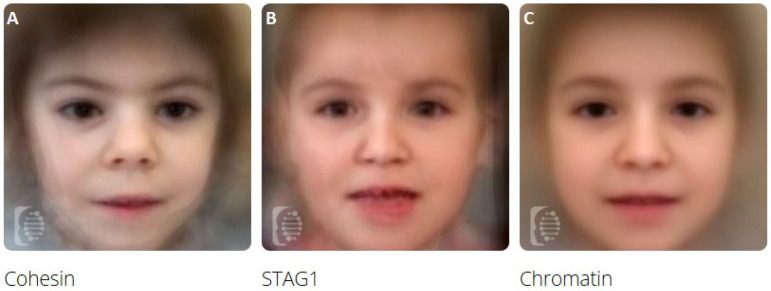
Composite images of the three groups of evaluated conditions: (**A**) Cohesinopathies, (**B**) *STAG1*, and (**C**) Chromatinopathies.

Thanks to published photographs, we were able to create a composite profile of the *STAG1*-related condition (N = 11, Figure 3B), and we compared this phenotype to the ones obtained, merging photos of patients affected by CoP (N = 10 photos) and CPs (N = 229) followed in our Center (see Figure 3A and Figure 3C, respectively).

Multiple comparisons of these profiles through F2G artificial intelligence (AI), reported in Table 3, resulted in a significant resemblance of MRD47 to CPs (false positive rate of 0.83).

Furthermore, we performed paired binary comparison among *STAG1*’s, CoPs’ and CPs’ photos, where complete results can be found in Table 4.

**Figure 4:**
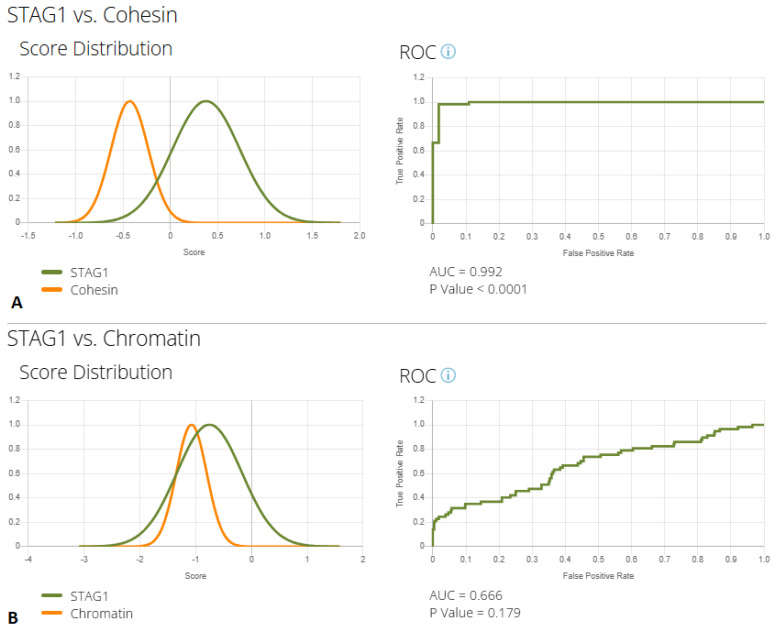
Results of binary comparison performed through the Face2Gene tool between MRD47 (green line) and Cohesinopathies (yellow line in (**A**)) and Chromatinopathies (yellow line in (**B**)). The ROC curve of the analysis and its relative *p* value are reported beside each comparison.

As these are exploratory analyses, the results have certain inevitable limitations due to the biased nature of the dataset, the variable image quality, and the small number of photos available, especially for MRD47. Still, despite these limitations, the results of these analyses support a divergence between the MRD47 and the CoP profiles, with a true positive rate of nearly 100% (see ROC curve in Figure 4A). Conversely, a much more significant overlap was detected by AI when comparing the *STAG1* phenotype to CPs (Figure 4B), suggesting that this specific condition should be numbered among CPs rather than CoPs.

## 5. Discussion

Once left behind the old “one gene-one syndrome” pillar, clinical geneticists are now sailing through difficult waters when attempting to properly classify genetic conditions. In this perspective, novel and rare disorders represent a unique opportunity to deepen our knowledge and eventually fill an empty space in such a complex puzzle. In particular, CPs represent one main challenging ground, given their expanding number and their overlapping and often mild phenotypes [22]. On the other hand, CoPs are generally considered more severe conditions presenting with multiple malformative pictures [7]. Driven by our experience with clinical and gestaltic diagnosis of CPs [22], we report a 10-year-old girl harboring a novel pathogenic variant in the *STAG1* gene, consistent with the diagnosis of a poorly known CoP. Spreading knowledge about this rare condition is crucial to advocate for laboratories to include the *STAG1* gene in their virtual panels when analyzing cases of suspected CPs or neurodevelopmental disorders. What caught our attention was the meaningful overlap of her clinical features with the phenotypes of CPs (e.g., late-onset childhood obesity and facial peculiarities). To expand present knowledge about MRD47 and deeply investigate the convergence between CoPs and CPs, we conducted a literature review of the *STAG1* phenotype. Only data from 27 individuals with point variants of this gene or with deletion encompassing exclusively *STAG1* were included. Given the well-known haploinsufficiency of this gene, our review has definitively demonstrated that missense variants in functional domains of *STAG1* are more likely to impact protein activity, with 9 out of 13 alterations localized in these regions (see Figure 2). Despite the limitations given by the small sample size, what emerges from our clinical review is that MRD47 is mainly a neurodevelopmental disorder, invariably associated with developmental delay and/or intellectual disability (present in 100% of individuals), often expressing into behavioral problems (reported in 40% of cases). Other neurological symptoms, such as epilepsy, anomalies in brain MRI, and autistic disorder, are reported in approximately 30% (see Table 2). Tentative genotype–phenotype correlations have already been described: in *STAG1*-deleted patients, features such as micro/brachycephaly and abnormal pregnancies appear to be more frequently reported [9,21]. It should be noted that, aligning with CoP growth patterns, nearly 10% of patients showed a reduced development during pregnancy; on the other hand, at older ages, growth anomalies appear to be common (mean prevalence of nearly 25%) but of variable expression: both the increase and reduction in head circumference and weight have been reported in this condition. In particular, weight gain has been reported in 4 out of 12 patients aged over 8 years, closely mirroring the pattern of weight gain observed in CP patients [23]. Other clinical manifestations appear to be variably present in this condition, including visual and auditory problems that affect less than ¼ of this population.

Skeletal and internal organ malformations are occasionally described (with a global prevalence below 30%), with sporadic reports of clubfeet, cleft lip and palate, and kidney anomalies (see Supplementary Material). According to collected data, the prevalence and severity of limb and heart defects in MRD47 significantly diverge from those described in CdL [7], aligning more with the presentation described for CPs [22]. Only one previous report has associated *STAG1* with external ear malformation and microtia [12], similarly to what has been frequently reported in *STAG2* patients (#MIM 301022). Given the limited number of collected cases, defining a clear genotype–phenotype correlation is still a challenging task. What we observed is that complete gene deletion might not always underlie more severe clinical pictures, as the case reported by Funato et al. [12]. In fact, even if no definitive conclusions can be drawn, available data suggest a higher prevalence of certain manifestations such as brain and skeletal abnormalities, joint hyperlaxity and cryptorchidism in patients harboring missense variants (see Supplementary Material). What still needs to be elucidated is the possible role of functional domains in defining clinical phenotypes associated with STAG genetic variants. From a biological perspective, the redundant role of STAG proteins [6] could explain the milder phenotypes observed in patients with complete loss of the STAG1 protein. Indeed, whilst the absent STAG1 protein can be readily replaced by STAG2, the presence of a defective protein—such as that found in missense variants—is likely to have a greater impact on the clinical presentation. Under this perspective, future research collecting a larger number of both *STAG1* and *STAG2* patients will help better delineate possible clinical differences related to impairment in specific functional domains.

The *STAG1*-related condition emerges from our review as characterized by a clinical presentation largely overlapping with CP phenotype, with features such as neurocognitive impairment, anomalies in hair distribution, and overgrowth ruling its clinical picture. Concerning dysmorphic features, *STAG1* profile emerges from our literature review as a quite aspecific phenotype, mainly characterized by anomalies in eyebrows, in eye/palpebral fissure morphology, and by abnormally wide mouth (see Supplementary Material). These facial details are also easily found in CPs, but relying solely on a list of peculiarities to infer a gestalt similarity is limited.

Therefore, to go beyond these observations, we applied F2G deep learning to quantitatively measure the similarities between MRD47 and both CoP and CPs. Considering single photo analyses, CdL emerges as the most recurrent suggested condition (see Supplementary Material). On the other hand, a CP was suggested among the top 10 conditions in one third of cases. Through this tool, we were able to create composite images of these three groups of conditions (see Figure 3), confirming to a well-trained clinical eye a strong resemblance between *STAG1* disorder and CP profile, emerging as a junction ring between cohesin complex and epigenetic machinery defects. The conducted research analysis, although constrained by the inherent limitations of this analytical tool, further confirmed our impressions: *STAG1* profile strongly overlaps with the one of CPs, with a high misclassification rate (83%, see Table 3) and a low accuracy (AUC = 0.66, see Figure 4B). On the other hand, *STAG1* and CoP profile can be easily distinguished by AI (AUC: 0.99, in Figure 4A).

Our case report, corroborated by F2G analyses, further strengthens the connection between CoPs and CPs. Although these two families of disorders have been traditionally distinct, the affinity between CPs and CoPs is a known but underexplored theme [24,25,26].

Although many biological mechanisms remain unclear, what emerges from recent studies is that STAG1 plays a key and independent role in modulating chromatin expression. According to these studies, STAG1 creates a specific form of cohesin module that is able to bind to chromatin loops even in the absence of *NIPBL* [6] and whose stability seems to be enhanced by SMC3 acetylation [27]. This unique and independent role in chromatin remodeling could represent the beginning of a link between STAG and diseases of the epigenetic machinery.

Before STAG1, a similar role of connectors between CPs and CoPs has also been recently suggested for ANKRD11 protein that binds to the cohesin complex with its N-terminal region, while the C-terminal one recruits the Histone Deacetylase 3 complex [28]. Furthermore, the CoP pathomechanism mainly lies in the dysregulation of gene expression rather than errors in chromosome segregation [21,29].

Embracing these affinities, CoP episignatures have recently started to emerge, and in some cases they partially overlap with CP ones [30,31,32,33,34]. Despite the limitations given by the small number of available cases, the present case report and literature review, together with the results of our gestaltic analysis, further tighten the junction between CoPs and CPs, suggesting the need to gather these historically distinct families of disorders. Future efforts will aim to determine the role of STAG proteins in the epigenetic machinery and to better define possible genotype–phenotype correlations mainly ruled by the affected protein domain rather than a defective single gene.

## 6. Conclusions

Starting from the report of a novel case of *STAG1*-related disorder, we performed a literature review of the clinical manifestation associated with these poorly known CoPs. Through AI technologies, we objectivated the strong phenotypical overlap of *STAG1* facial features to the profile of epigenetic disorders. The present work further strengthens the link between CoPs and CPs, which should nowadays be considered a continuous spectrum of neurodevelopmental disorders driven by imbalances in chromatin modulation.

## Figures and Tables

**Table 1:** Details of pathogenic or likely pathogenic single-nucleotide variants of *STAG1*. Abbreviations: c.: coding DNA, dn: de novo, het: heterozygous, ID: patient identity, Inher.: inheritance, mat: maternal, NA: not available; Pt: patient, Zig.: zygosity.

ID	*STAG1* (NM_005862.3)	NP_005853.2	Inher.	Zig.
Lehalle et al., Pt 4 [9]	Chr3: 136109538–136310711	p.0?	NA	het
Lehalle et al., Pt 5A and 5B [9]	Chr3: 136254742–136427833	p.0?	mat	het
Seymour et al., 2024 [15]	c.17T>G	Leu6Ter	dn	het
**Present case**	**c.89_90delAG**	Glu30Glyfs*18	**dn**	**het**
Jiang et al., 2025 [21]	c.500dup	Gly168Trpfs*13	dn	het
Lehalle et al., Pt 7 [9]	c.641A>G	Gln214Arg	dn	het
Safgren 2024 [16]	c.643G>A	Val215Ile	dn	het
Lehalle et al., Pt 9 [9]	c.646A>G	Arg216Gly	dn	het
Lehalle et al., Pt 12 [9]	c.659A>G	His220Arg	dn	het
Funato et al., 2021 [12]	c.901C>T	Arg301Cys	NA	het
Lehalle et al., Pt 13 [9]	c.997A>C	Lys333Gln	dn	het
Xie et al., 2025 [18]	c.1027-2A>G	Gln343Alafs*19	dn	het
Lehalle et al., Pt 15 [9]	c.1052T>G	Leu351Trp	dn	het
Lehalle et al., Pt 10 [9]	c.1118G>A	Arg373Gln	dn	het
Yuan et al., 2019, Pt 2 [14]	c.1129C>T	Arg377Cys	dn	het
Bregvadze et al., 2024 [11]	c.1183C>T	Arg395*	dn	het
Cipriano et al., 2024 (2 Pt) [10]	c.1279G>A	Val427Ile	dn	het
Lehalle et al., Pt 8 [9]	c.1433A>C	His478Pro	dn	het
Lehalle et al., Pt 11 [9]	c.1460_1464dup	Trp489Valfs*10	dn	het
Lehalle et al., Pt 16 [9]	c.1736dup	Ser580Valfs*21	dn	het
Yuan et al., 2019, Pt 1 [14]	c.2009_2012del	Asn670Ilefs*25	dn	het
Serban et al., 2025 [17]	c.2116del	Asp706Ilefs*15	dn	het
Zhang et al., 2025 [20]	c.2549G>A	Gly850Asp	dn	het
Di Muro et al., 2021 [13]	c.2769_2770del	Ile924Serfs*8	dn	het
Lehalle et al., Pt 14 [9]	c.2936A>G	Lys979Arg	dn	het

**Table 2:** Clinical manifestations reported in patients affected by *STAG1* pathogenic alterations. Abbreviations: abn.: abnormalities, ASD: autism spectrum disorder, DD: developmental delay, dis.: disorder, GERD: gastroesophageal reflux disease, GU: genitourinary, HC: head circumference, ID: intellectual disability, MRI: magnetic resonance imaging, SD: standard deviations, SGA: small for gestational age.

Feature	N	Percentage
Male	14/27	52
Prenatal abn.	5/26	19
SGA	3/27	11
Hypotonia	4/26	16
Feeding diff.	11/27	41
Infantile GERD	3/26	11
DD	26/27	96
ID	17/17	100
ASD	5/19	26
Behavioral abn.	8/20	40
EEG abn./Seizure	8/22	36
Brain MRI abn.	7/25	28
Neurological abn.	4/25	16
Eye abn.	5/26	19
Ear abn.	4/26	15
HC >2 SD	1/25	4
HC <2 SD	4/25	16
Height <2 SD	5/27	18
Height >2 SD	1/27	4
Weight >2 SD	4/27	15
Weight <2 SD	4/27	15
Hyperlaxity	5/26	19
Hand/Foot abn.	9/26	35
Skeletal abn.	7/26	27
Heart abn.	0/25	0
Cryptorchidism	3/14	12
Other GU abn.	1/25	4
Constipation	2/24	8
Sleep dis.	2/25	8
Endocrine abn.	3/25	12
Recurrent Infections	3/25	12
Hypertrichosis	3/25	12
Teeth abn.	4/26	15

**Table 3:** A confusion matrix obtained through multiple-comparison analysis performed among the three mentioned classes by F2G deep learning technology. Bold numbers represent the true positive rate. Abbreviations: CoPs: Cohesinopathies, CPS: Chromatinopathies.

	Predicted		
**Actual**	***STAG1***	**CoPs**	**CPS**
***STAG1***	**0.17**	0.00	0.83
**CoPS**	0.00	**0.39**	0.61
**CPS**	0.00	0.00	**1.00**

**Table 4:** Complete results of binary comparison analyses between paired cohorts. Abbreviations: AUC: area under the curve, CoPs: Cohesinopathies, CPS: Chromatinopathies, STD: standard deviation.

Binary Comparisons	Cases	Frontal Images	Mean AUC	AUC STD
***STAG1*** ** vs. CoPs**	10 vs. 9	10 vs. 11	1.00	0.00
***STAG1*** ** vs. CPS**	10 vs. 209	10 vs. 282	0.67	0.11
**CoPs vs. CPS**	9 vs. 209	10 vs. 282	1.00	0.00
**CPS vs. All Other Cohorts**	209 vs. 20	282 vs. 22	0.77	0.07
**CoPs vs. All Other Cohorts**	9 vs. 220	10 vs. 293	0.99	0.01
***STAG1*** ** vs. All Other Cohorts**	10 vs. 218	10 vs. 293	0.73	0.07

## Data Availability

Data analyzed in this study are available for consultation from the corresponding author under reasonable request.

## Supplementary Materials

The following supporting information can be downloaded at https://www.mdpi.com/article/10.3390/genes17040483/s1, Excel file containing data used for the present analysis: sheet 1: clinical presentation of all reported patients, Sheet 2: results of F2G analysis, Sheet 3: STAG1 reported dysmorphisms.

## Abbreviations

The following abbreviations are used in this manuscript:

CPs	Chromatinopathies
CoPs	Cohesinopathies
MRD47	Intellectual Developmental Disorder, Autosomal Dominant 47
ID	Intellectual Disability
HC	Head Circumference
SD	Standard Deviation
NA	Not Available
SGA	Small for Gestational Age
EEG	Electroencephalogram
MRI	Magnetic Resonance Imaging
